# Method development and validation for the extraction and quantification of sesquiterpene lactones in *Dolomiaea costus*

**DOI:** 10.1016/j.ultsonch.2024.107128

**Published:** 2024-10-26

**Authors:** Mohammed Aldholmi

**Affiliations:** Department of Natural Products, College of Clinical Pharmacy, Imam Abdulrahman Bin Faisal University, Dammam 31441, Kingdom of Saudi Arabia

**Keywords:** Costunolide, Dehydrocostus lactone, Qust, Kuth, Ultrasonic bath, Ultrasonic homogeniser

## Abstract

*Dolomiaea costus*, commonly known as Indian costus, is a medicinal plant from the Asteraceae family. The root and powder of costus have been widely used to treat various health conditions. The primary bioactive compounds in this plant are sesquiterpene lactones, particularly costunolide and dehydrocostus lactone. This study aimed to establish a rapid, environmentally friendly, and cost-effective method for the high-throughput extraction and quantification of sesquiterpene lactones in Indian costus. Ultrasonic bath (UB) and UPLC/MS-MS were employed to extract and analyse 49 Indian costus samples. Aqueous ethanol was identified as the most effective solvent system for extracting and analysing sesquiterpene lactones. The extraction efficiency of the ultrasonic bath was comparable to that of the ultrasonic homogeniser while shaking showed the lowest efficiency. The environmentally friendly UPLC/MS-MS analysis revealed mean concentrations (±SD; μg/100 μg) of 1.00 (±0.39) for costunolide and 0.70 (±0.25) for dehydrocostus lactone. An inverse correlation was observed between sesquiterpene lactone content and sample colour. Most samples contained costunolide levels above the minimum limit (0.6 %) specified by the Chinese monograph, but only a few met the 1.8 % threshold for total sesquiterpene lactones. Given the importance of bioactive sesquiterpene lactones for medicinal efficacy, insufficient levels may result in diminished therapeutic value. Therefore, standardising Indian costus products is crucial to ensure quality and appropriate dosing. This study contributes to the standardisation of Indian costus, a vital step towards ensuring the efficacy and safety of herbal products.

## Graphical abstract

**Figure:**
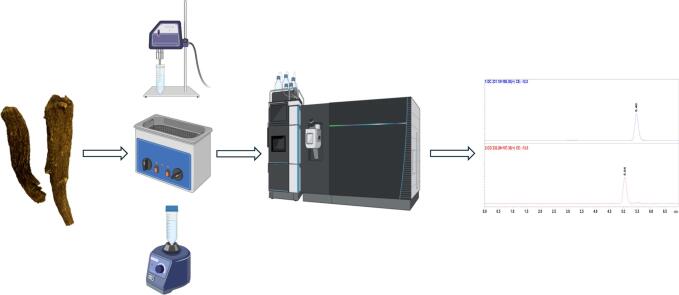

## 1 Introduction

**Fig. 1:**
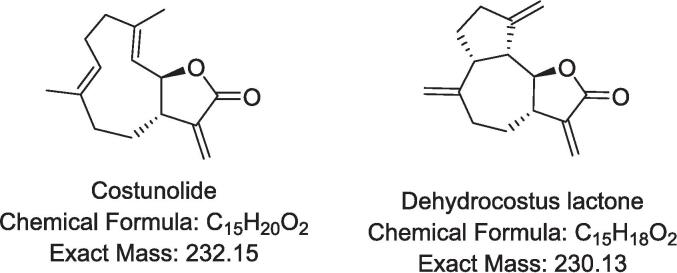
Chemical structure, molecular formula, and exact mass of costunolide and dehydrocostus lactone.

*Dolomiaea costus,* formerly known as *Saussurea costus* and *Aucklandia costus*, is a medicinal plant belonging to the Asteraceae family [1]. It is native to the Himalayan regions of India, Pakistan, and China [2]. *Dolomiaea costus* is commonly known as Indian costus, Mu Xiang, costus, Kuth, or Qust [3]. The root has been used for medicinal purposes since ancient times by the Greeks and Arabs and was introduced to Europe by Arab physicians [3]. Costus root and its powder are employed to treat numerous health conditions, including infectious diseases, liver disorders, inflammation, gastritis, diarrhoea, ulcers, allergies, asthma, coughs, epilepsy, joint pain, scalp scabies, and blood-related disorders [2]. The root or powder is taken orally with warm water for gastrointestinal issues, while root paste or oil is applied topically to alleviate skin inflammation and joint pain [2]. The broad medicinal applications of Indian costus are attributed to the presence of various bioactive secondary metabolites, including sterols, flavonoids, coumarins, phenylpropanoids, lignans, alkaloids, monoterpenes, triterpenes, sesquiterpenes, and sesquiterpene lactones [4]. The key bioactive sesquiterpene lactones in costus root oil and extract are costunolide and dehydrocostus lactone (Fig. 1).

Several varieties of Indian costus are used in traditional medicine systems, including Ayurveda, Unani, Siddha, Arabian and Traditional Chinese Medicine [2], [3]. Dioscorides, often referred to as the father of pharmacognosy, described the best Indian costus as “fresh, light-coloured, compact and of firm texture, dry, not worm-eaten, devoid of an acrid smell, and tasting hot and biting” [3]. This has led to the common belief that the light-coloured variety of Indian costus is more effective than the darker varieties. However, the correlation between the colour of Indian costus and its bioactive constituents remains unexplored. Indian costus is typically sold in herbal shops in two forms: root or powder. The bioactive compounds in the powdered form may be prone to degradation, particularly when stored for extended periods after grinding. A significant instability of total sesquiterpenes has been observed, with around 20 % of total sesquiterpenes lost 15–20 days after the powdering process [5]. This considerable loss of bioactive constituents underscores the need for proper storage and handling of Indian costus to preserve its bioactivity. The Chinese monograph for the dried herbal drug of Indian costus specifies a minimum content of 0.6 % for costunolide (CO) and a minimum of 1.8 % for the sum of costunolide and dehydrocostus lactone (DC) [5]. Although this herbal drug is widely consumed in Saudi Arabia and other Arab countries as part of traditional medicine, there are no specific regulations or quality standards to ensure its efficacy. Moreover, the Indian costus samples marketed in Saudi Arabia are assumed to be effective but have not been evaluated for their minimum CO and DC content.

To ensure the high quality and efficacy of Indian costus, samples of the root or powder should be analysed for the minimum levels of sesquiterpene lactones, particularly costunolide (CO) and the combined content of costunolide and dehydrocostus lactone (CO + DC). Several analytical methods have been reported for quantifying CO and DC, including densitometric thin-layer chromatography [6], HPLC-UV [7], HPLC-PDA [8], [9], [10], and UPLC-DAD/MS-MS [11]. However, these methods have various disadvantages, such as the use of toxic solvents, long analysis times with high flow rates (making them time-consuming and economically inefficient), low sensitivity, and poor reproducibility. Densitometric thin-layer chromatography is a cost-effective method for the simultaneous quantification of CO and DC [6]. However, it employs toluene, a solvent that is harmful to both humans and the environment. Furthermore, this method is impractical for analysing multiple samples due to the limited space on thin-layer chromatography plates and the potential for band overlap. HPLC-UV and HPLC-PDA methods have also been developed for CO and DC quantification [7], [8], [9], [10], but they have limitations, including the use of toxic solvents like methanol and acetonitrile, a high flow rate of 1 mL/min, and extended analysis times (15–35 min), which make them inefficient in terms of time and cost. Additionally, the detection and quantification limits of UV and PDA detectors are higher (μg/mL) compared to the more sensitive MS detector (μg/L). A UPLC-PDA/MS-MS method has been developed for the quantification of sesquiterpene lactones, but it also involves toxic solvents such as methanol and acetonitrile [11]. Moreover, while the MS detector was used for compound identification, quantification was still carried out using the less sensitive PDA detector. Therefore, there is a need for an eco-friendly, safe, sensitive, and cost-effective method for the quantification of CO and DC in Indian costus.

Previously reported methods for extracting costunolide (CO) and dehydrocostus lactone (DC) have several drawbacks, including the use of toxic or costly solvents, long extraction times, multiple steps, and the potential degradation of bioactive compounds due to heating. Conventional solvent extraction methods used for Indian costus are lengthy, multistep processes that can take several days and require large volumes of solvents[11]. While Soxhlet extraction or heating under reflux shortens the process (4–4.5 h), the high temperatures involved can degrade bioactive compounds during extraction [6], [8]. Moreover, heating poses a significant environmental impact, mainly due to high energy consumption, leading to increased carbon emissions, resource depletion, and air pollution. In contrast, ultrasonic extraction methods offer several advantages over traditional solvent extraction, particularly in terms of efficiency, environmental impact, and scalability. Sonication using an ultrasonic homogeniser (UH) [12], [13], [14] or ultrasonic bath (UB) (UB) [15], [16] has been successfully applied to the extraction of various herbal products. Ultrasonic waves disrupt cell walls, accelerate solvent penetration, and enhance mass transfer, resulting in higher yields compared to traditional methods [17]. While both UH and UB utilise high-frequency sound waves, they differ in how energy is transferred to the sample [17]. UH uses a power generator, transducer, amplifier, and a probe that is immersed directly in the sample, transferring ultrasonic energy directly to the particles. On the other hand, UB uses a power generator, transducer, and bath to produce ultrasonic energy that diffuses across a liquid medium, impacting the sample immersed in the bath. Although UH has high extraction efficiency, it can only process one sample at a time, making it unsuitable for high-throughput extraction of costus samples. The frequent immersion of the ultrasonic probe into multiple samples carries a high risk of cross-contamination, which can affect the accuracy of quantification.

While sonication extraction methods for Indian costus exist, they still depend on either toxic solvents, such as methanol and chloroform, or expensive solvents, like limonene.[9], [10]. The environmental impact and toxicity of solvent systems play a crucial role in sustainable extraction processes. The selection of a solvent can greatly affect not only the extraction efficiency but also the safety and environmental sustainability of the process. While organic solvents like hexane, toluene, benzene, and chloroform are efficient, their associated environmental and health risks are prompting researchers and industries to seek safer, more environmentally friendly alternatives [18]. Solvents like hexane and benzene are highly toxic to humans and resistant to biodegradation, posing long-term environmental risks [18]. Methanol is a commonly used solvent but is highly toxic and can cause severe health issues, including neurological damage and blindness [19]. Acetone is less toxic than methanol, although it can still be hazardous in high concentrations [20]. However, acetone is a volatile organic compound (VOC) and may contribute to air pollution and the formation of ground-level ozone, a harmful pollutant [18]. Green solvents such as water and ethanol are gaining attention due to their lower toxicity, reduced environmental impact, and alignment with the principles of green chemistry. Compared to other solvents, water is the safest and most environmentally friendly option available, but its use is limited to extracting polar compounds. On the other hand, ethanol is considered relatively safe for human health and is widely used in the food, pharmaceutical, and cosmetic industries. Compared to petroleum-based solvents, ethanol has a lower environmental impact, minimal toxicity, and evaporates easily, reducing long-term environmental concerns. An ethanol–water mixture is more environmentally favourable compared to using pure ethanol [18].

Due to the limitations of previous extraction and analysis methods mentioned above, this study aimed to develop a fast, green, sensitive and cost-effective extraction and analysis method for the high-throughput extraction and quantification of sesquiterpene lactones from Indian costus. Various chromatographic and mass spectrometry (MS) conditions are tested to establish an efficient method for quantifying CO and DC in Indian costus. The extraction efficiency of different solvents (50 % ethanol, absolute ethanol, methanol, acetone, and water) and extraction methods (UH, UB, and vortexing) is investigated to shorten the extraction time while using non-toxic solvents. The ultimate objective is to apply the developed extraction and analysis method practically for quantifying CO and DC in Indian costus root and powder samples collected from the Saudi market, thereby evaluating the overall quality and potential efficacy of the marketed products. Additionally, this study determines, for the first time, the correlation between sample colour and sesquiterpene lactone content.

## 2 Materials and methods

### 2.1 Solvents and Chemicals

HPLC-grade solvents (methanol, ethanol, and acetone) and LCMS-grade ethanol were purchased from Sigma-Aldrich (Darmstadt, Germany). High-purity grade (≥ 98 %) costunolide (CO) and dehydrocostus lactone (DC) were purchased from UFC Biotechnology (Buffalo, New York, United States). A Pure Lab Ultra water system (ELGA, High Wycombe, UK) was utilised to prepare purified water.

### 2.2 Samples collection, authentication, and preparation

Indian costus powder (30) and root (19) samples were purchased from different herbal shops in the Eastern Province of Saudi Arabia and authenticated in the natural products lab via macroscopic and microscopic inspection. The roots were thoroughly cleaned and cut into small pieces. All samples were powdered using an electric blender and passed through a stainless-steel sieve with different aperture sizes (0.5 mm, 1.0 mm, and 1.4 mm; Laboratory Test Sieve, BS 410–1, Endecotts Ltd., London, England). The powdered samples (1.0 mm particle size) were coded from C1 to C49 and used to extract and quantify sesquiterpene lactones. The colour of the powdered samples was recorded as either light brown or dark brown.

### 2.3 UPLC-MSMS method development and validation

The standard stock solutions (10 mg/L) for each sesquiterpene lactone were prepared in ethanol, and a combined stock solution (10 mg/L) was created for both standards. This stock solution was successively diluted to achieve a linearity range of 5–500 μg/L (5, 10, 25, 50, 100, 200, and 500 μg/L). The diluted standard solutions were filtered through a syringe filter (0.2 μm) prior to analysis. The Nexera X2 UHPLC (Shimadzu, Japan), connected to a Shimadzu 8050 triple quadrupole mass spectrometer, was used for UHPLC-MS/MS analysis, with LabSolutions software V 5.93 (Kyoto, Japan) for instrument control and data processing.

Chromatographic separation was optimised on a Shim-pack XR-ODS Column (75 mm, 3 mm, 2.2 µm, Shimadzu, Japan) using various isocratic and gradient ratios of the mobile phase (water as solvent A and ethanol as solvent B), a flow rate of 0.1–0.5 mL/min, column temperatures ranging from 20 to 50 °C, and a sample injection volume of 1–5 µL. For mass analysis, the conditions were as follows: the temperatures of the ESI (electrospray ionisation) interface and the desolvation line were set at 300 °C and 250 °C, respectively, while the heat block temperature was maintained at 400 °C. The flow rate of the nitrogen nebulising gas was 3 L/min, whereas the flow rates for the drying gas and air heating gas were both set at 10 L/min. The UHPLC retention time and the MRM (multiple reaction monitoring) transitions of the MS fragments were used to identify the target analytes. Upon identification, target analytes were quantified using the highest-intensity fragment ion. The accuracy (%), limit of detection (LOD), relative standard deviation (%RSD), and other MS parameters were calculated for each compound by LabSolutions software using various concentrations.

### 2.4 Development of solvent system and sonication method

An amount of 300 mg of costus powder was added to 50 mL tubes containing 30 mL of 50 % ethanol, absolute ethanol, methanol, acetone, or water. Subsequently, the samples were extracted using an ultrasonic homogeniser (UH) comprising a 20-kHz (50 Watt) ultrasonic processor (Fisher Scientific, 2000 Park Lane, Pittsburgh, PA, USA) attached to a transducer (Model CL-334), a fixed horn (220-A), and a removable titanium probe (420-A; 1 mm diameter). The UH parameters for extraction were set as previously described for the extraction of Glycyrrhiza glabra powder [12]. Briefly, each sample was extracted for 5 min with an amplitude of 40 % and a pulse time of 40 s. To compare the extraction efficiency of UH to that of the ultrasonic bath (UB), another 300 mg sample of costus powder was extracted with 50 % ethanol for 5 min using a Branson UB featuring a 40 kHz rugged industrial transducer (Connecticut, USA). A third sample of costus powder was shaken for 5 min using a Corning® LSE™ vortex mixer (New York, USA) to compare ultrasound-assisted extraction with conventional solvent extraction (vortex). All samples were centrifuged (Kubota Corporation, Japan) at 3600 rpm for 10 min to separate the powder residues from the liquid extract. An aliquot of 3 µL from each sample was diluted to a final volume of 1 mL and filtered through a syringe filter (0.2 μm). The filtered solutions from each experiment were analysed using the developed UPLC-MS/MS method.

### 2.5 Application of UB extraction method for high-throughput extraction of costus

The developed fast and cost-effective ultrasonic bath (UB) extraction method was applied to the 49 costus samples. An amount of 10 mg of costus powder was added to 1.5 mL centrifuge tubes containing 1 mL of 50 % ethanol and extracted using the UB extraction method. The samples were centrifuged at 13,000 RPM (Heraeus Biofuge Pico, New York, United States) for 10 min to separate the powder residues from the liquid extract. An aliquot of 1 µL from each sample was diluted to a final volume of 1 mL and filtered through a syringe filter (0.2 μm). The filtered solutions were analysed using the developed UPLC-MS/MS method.

### 2.6 Statistical analysis

SPSS Version 27.0 (IBM Corporation, Armonk, NY, USA) and GraphPad Prism 8 (GraphPad Software, Boston, MA, USA) were used to analyse the data and create the graphs. The results are expressed as the mean ± standard deviation (SD) from at least three independent experiments.

## 3 Results and Discussion

### 3.1 UPLC-MSMS method development and validation

**Fig. 2:**
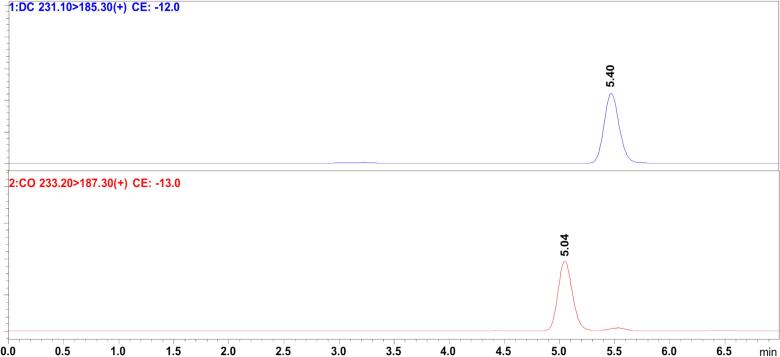
Chromatographic separation and ion transitions of CO and DC.

The development of the UPLC-MSMS method achieved optimal simultaneous chromatographic separation of CO and DC (Fig. 2) using isocratic elution with 70 % ethanol as the mobile phase, a flow rate of 0.15 mL/min, a column temperature of 40 °C, and a sample injection volume of 1 µL. The sesquiterpene lactones were effectively separated with a non-toxic green solvent system at a low flow rate within 7 min, with retention times of 5.04 and 5.40 min for CO and DC, respectively.

The optimised MS conditions for the detection and quantification of CO and DC were established in positive ion mode, with [M − H]^+^ precursors observed at *m*/*z* 233.2 for CO, showing a main fragment ion at *m*/*z* 187.3, and at *m*/*z* 231.1 for DC, with a main fragment ion at *m*/*z* 185.3. The optimal collision energy (CE) for the analytes was determined by evaluating the fragmentation pattern and peak shape of the most abundant fragments, resulting in CE values of −13 for the transition 233.20 > 187.30 and −12 for 231.10 > 185.30. The MS parameters related to accuracy, limit of detection (LOD), limit of quantification (LOQ), and other relevant metrics are summarised in Table 1, demonstrating the high precision and accuracy of the developed method for the detection and quantification of CO and DC.

**Table 1:** MS parameters for the optimised quantitative method.

Parameters	CO	DC
Linear range (μg/L)	5–500	5–500
*m*/*z*	233.20 > 187.30	231.10 > 185.30
Regression equation	Y = (21301.4)X + (−36303.2)	Y = (25122.8)X + (−31447.4)
Correlation coefficient (r^2^)	0.999	0.999
Accuracy (%±SD)	108.2 (±17.9)	107.0 (±16.3)
Relative Standard Deviation (%RSD)	7.8	8.3
LOD (μg/L)	1.28	2.01
LOQ (μg /L)	3.86	6.09

To the best of our knowledge, this is the first environmentally friendly and cost-effective analytical method developed for detecting and quantifying CO and DC in Indian costus. Previous analytical methods have been reported, but they have several disadvantages and limitations. For instance, Vijayakannan et al. developed a cost-effective method using densitometric thin-layer chromatography for the simultaneous quantification of CO and DC; however, this method employs toluene, a harmful solvent detrimental to both human health and the environment [6]. Furthermore, the limited space on thin-layer chromatography plates poses challenges for analysing multiple samples, as it may lead to band overlap. Kang et al. reported an HPLC-UV quantitative method for determining costunolide, which utilised 65 % methanol as an eluent with a flow rate of 1 mL/min.[7]. Costunolide eluted at a retention time of 9.5 min, making this method unsuitable for rapid analysis, especially since it was designed for the quantification of a single sesquiterpene lactone. Other studies have also documented HPLC-PDA analysis methods for the simultaneous determination of CO and DC using isocratic elution with distilled water and acetonitrile [8], [9]. However, these methods are limited by the use of acetonitrile (a non-green solvent obtained as a by-product of the production of acrylonitrile [21]). They also used a high flow rate of 1 mL/min and extended time (15–20 min) of analysis, which are costly, time-consuming and environmentally unfriendly. Similarly, another study reported an HPLC-PDA analysis method employing methanol, acetonitrile and 0.5 % phosphoric acid as the mobile phase with a 1 mL/min flow rate and a detection time of 35 min [10].

A UPLC-PDA/MS-MS quantitative method for determining sesquiterpene lactones has been developed; however, it involves toxic solvents, particularly methanol and acetonitrile [11]. Moreover, the MS detector was only employed for compound identification, while quantification was performed using the less sensitive PDA detector. In the current study, MS-MS has been successfully utilised for the detection and quantification of CO and DC in Indian costus extracts, achieving high sensitivity and low detection and quantification limits. The LOD values for costunolide (1.28 μg/L) and dehydrocostus lactone (2.01 μg/L) are lower than those reported in previous studies, which ranged from 60 to 1500 μg/L for costunolide and from 110 to 1300 μg/L for dehydrocostus lactone [7], [8], [9], [10]. Similarly, the LOQ values for costunolide (3.86 μg/L) and dehydrocostus lactone (6.09 μg/L) are also lower than those documented in earlier studies, which ranged from 210 to 4600 μg/L for costunolide and from 370 to 4000 μg/L for dehydrocostus lactone [7], [8], [9], [10]. The accuracy and precision values fall within acceptable limits and are comparable to those reported in previous research [7], [8], [9], [10]. Hence, our green quantitative method meets the criteria for an eco-friendly, safe, sensitive, and cost-effective approach to quantifying CO and DC in Indian costus. However, the current method is limited to the quantification of CO and DC, while other sesquiterpene lactones have been shown to contribute to the overall biological activity of Indian costus [3]. Moreover, the developed method excludes compounds from other chemical classes, such as tannins, alkaloids, flavonoids, and anthraquinones, identified in Indian costus[2]. Therefore, a comprehensive analytical method for the simultaneous quantification of multiple phytoconstituents in Indian costus presents an opportunity for enhancing the quality and standardisation of this herbal drug. The main challenge in developing such a method will be the analysis time, which is proportional to the number of analytes.

### 3.2 Impact of the solvent system on sesquiterpene lactones

**Fig. 3:**
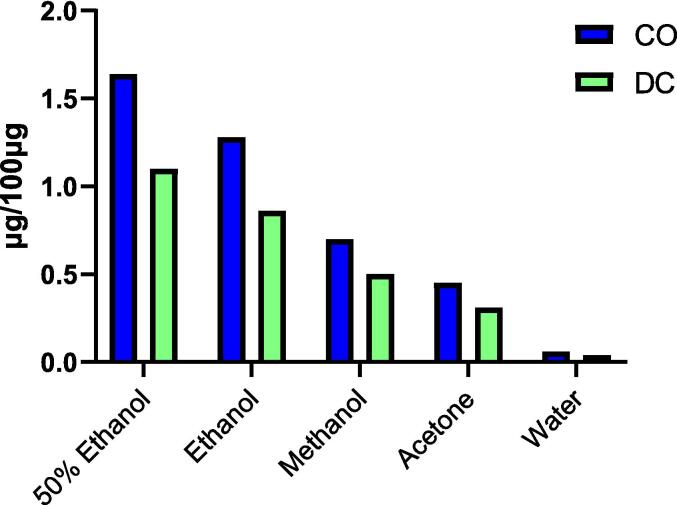
The impact of the solvent system on the CO and DC quantity in the extract.

Our research involved a thorough comparative evaluation of the efficiency of various solvent systems for the extraction of CO and DC (Fig. 3). The results revealed that 50 % ethanol emerged as the most efficient solvent system, followed by absolute ethanol. Water, while the least efficient solvent system on its own, appeared promising when combined with ethanol. Among the investigated organic solvents, acetone had the lowest extraction efficiency. On the other hand, methanol demonstrated higher extraction efficiency than acetone but lower than both aqueous and absolute ethanol. Rao Vadaparthi, P.R. et al. reported methanol as the most efficient solvent system for the extraction of CO and DC from Indian costus compared to chloroform, hexane, and ethyl acetate [9]. However, less toxic solvents such as ethanol, aqueous ethanol, and water have not been compared to the more toxic solvents. Therefore, this study reveals the superiority of ethanol and aqueous ethanol over methanol for extracting bioactive compounds from Indian costus. While ethanol has a lower boiling point, making it easier to evaporate, aqueous ethanol is more effective for extracting both CO and DC. Additionally, the aqueous ethanol solvent system is more cost-effective and environmentally friendly due to the presence of water. Hence, 50 % ethanol was employed for extraction in the subsequent experiments.

### 3.3 Impact of sonication method on sesquiterpene lactones

**Fig. 4:**
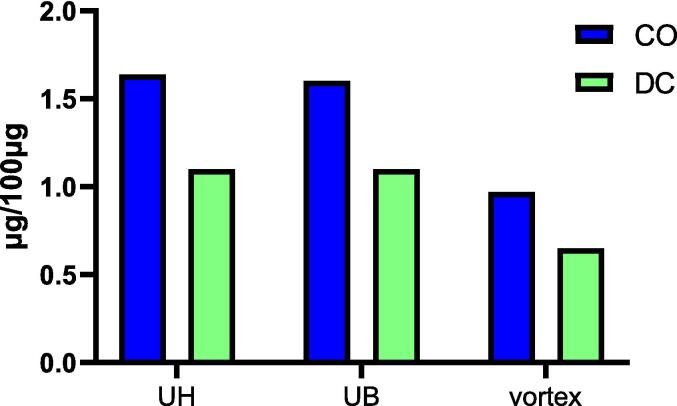
The impact of the sonication method on the CO and DC quantity in the extract.

We previously established and optimised UH parameters (extraction time of 5 min, amplitude of 40 % and pulse time of 40 sec) to extract *Glycyrrhiza glabra* powder [12]. This method was subsequently applied to extract Indian costus, demonstrating high efficiency in extracting CO and DC. Compared to traditional solvent extraction methods, ultrasonic extraction techniques offer shorter extraction times, reduced solvent and energy consumption, improved yields, and better preservation of sensitive compounds. Although the UH device provides high extraction efficiency, it is limited to processing one sample at a time, making it unsuitable for high-throughput extraction of costus samples. Additionally, the frequent immersion of the ultrasonic probe into multiple samples poses a significant risk of cross-contamination, potentially impacting quantification accuracy. In contrast, the main advantage of the UB extraction method is its capacity to simultaneously extract multiple samples due to its larger capacity. This study compared UH with UB and vortex extraction methods to identify the optimal technique for extracting multiple costus samples within 5 min. The comparison revealed that both UH and UB yielded similar amounts of CO and DC, while the vortex extraction method produced significantly lower quantities (Fig. 4).

Previous studies have employed various extraction methods that required lengthy extraction times and multiple steps. For instance, the conventional solvent extraction of Indian costus with 95 % ethanol involved seven extraction steps, each lasting 24 h at room temperature [11]. In another study, Indian costus was extracted in a shorter time of 4 h using a Soxhlet apparatus for hot extraction with methanol as the solvent [6]. Similarly, extraction with 70 % methanol under reflux was conducted for three sessions of 90 min each [8]. However, the heating involved in both the Soxhlet and reflux methods can lead to the degradation of bioactive compounds during extraction. Rao Vadaparthi, P.R. et al. developed a sonication extraction method that takes 30 min, but the primary drawback of this approach is the use of toxic solvents such as methanol and chloroform [9]. Another study employed a 250 W ultrasonic system for 30 min to extract costunolide (CO) and dehydrocostus lactone (DC) from Indian costus, using limonene as the extraction solvent [10]. While limonene is considered a green solvent, it has several disadvantages, including fluctuating prices, difficulties in solvent removal due to its high boiling point (175–177 °C), limited water solubility, and potential skin and airway irritation [22]. Therefore, our developed ultrasound-assisted extraction method is rapid, environmentally friendly, and cost-effective, making it suitable for extracting CO and DC from Indian costus. This method may also be applied to extract other sesquiterpene lactones from different natural sources. However, factors such as solvent concentration, extraction time, and temperature may significantly influence the extraction of sesquiterpene lactones. Therefore, further studies are needed to assess how these parameters affect extraction efficiency.

### 3.4 UB extraction and analysis of Indian costus samples

#### 3.4.1 Descriptive analysis of CO and DC in Indian costus

The descriptive analysis of the Indian costus samples (*N* = 49) revealed a mean (±SD; μg/100 μg) of 1.00 (±0.39) for costunolide with a sum of 49.10 μg/100 μg. The maximum individual concentration of costunolide observed in all samples was 2.04, while the lowest concentration recorded was 0.06 μg/100 μg. For dehydrocostus lactone, the mean concentration in the investigated samples was 0.70 (±0.25) μg/100 μg, with a total of 34.44 μg/100 μg. The maximum and minimum amounts for DC were 1.43 and 0.25 μg/100 μg, respectively. The concentrations of CO and DC in most costus samples fall within the ranges reported in previous studies, which noted concentrations of CO from 0.057 to 1.9 μg/100 μg and DC from 0.12 to 2.1 μg/100 μg [7], [8], [9], [10]. This variation may be caused by several factors, including the origin and genotype of the samples, as well as storage, transport, and preparation conditions. Kang et al. [7] reported significant differences in the mean CO levels across five Indian costus samples available in Korea. Similarly, substantial variations in CO and DC concentrations were observed in Indian costus samples marketed in India [6]. Cultivated samples typically contained significantly higher amounts of these compounds compared to wild samples. The lowest reported concentrations were 0.057 μg/100 μg for CO and 0.12 μg/100 μg for DC, as found by Guangyu et al. in a sample collected from the Chinese market [10]. In contrast, Indian costus samples collected from the Indian market have shown concentrations of up to 1.9 μg/100 μg for CO and 2.1 μg/100 μg for DC [9]. However, these studies employed different solvent systems and extraction methods, which are critical factors affecting the extracted and quantified amounts of CO and DC. Consequently, the extraction method developed in this study can serve as a standard tool for high-throughput extraction and comparison of Indian costus samples available in the market.

**Fig. 5:**
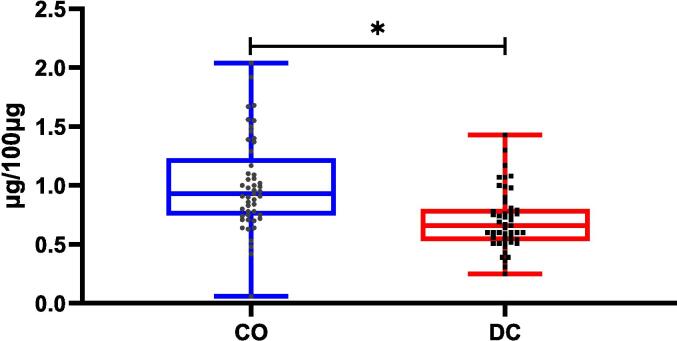
A box and whiskers plot of CO and DC in Indian costus samples.

The paired samples test (Table 2) and boxplot (Fig. 5) indicate a significant difference (*p* < 0.001) between the mean values of CO and DC in the investigated 49 Indian costus samples. On an individual basis, one sample (C2) contained substantially higher amounts of DC than CO (four times), while three samples (C31, C32, and C39) had approximately equal amounts of CO and DC. The remaining samples exhibited significantly higher amounts of CO than DC. Although most previous quantification studies have reported DC as the major sesquiterpene lactone in Indian costus [7], [8], [9], [10], one study has revealed higher amounts of CO than DC in some Indian costus samples [5]. Furthermore, most isolation and purification studies have yielded higher CO concentrations compared to DC from Indian costus [23], [24]. The CO to DC ratio may vary based on the origin of the extracted sample, extraction method, and preparation and analysis time. It has been reported that the CO content in dried extracts can decrease by 24 % within seven days post-extraction [7]. Therefore, quantitative analysis should be conducted immediately after extraction to ensure accurate determination of CO and DC levels.

**Table 2:** Paired samples test of CO and DC in Indian costus.

Paired Samples Test									
		Paired Differences					t	df	Sig. (2-tailed)
		Mean	Std. Deviation	Std. Error Mean	95 % Confidence Interval of the Difference				
					Lower	Upper			
Pair 1	CO − DC	0.29918	0.19090	0.02727	0.24435	0.35402	10.971	48	<0.001

#### 3.4.2 Concentrations of CO and CO + DC in powder vs. Root samples

A previous study reported significant instability in total sesquiterpenes following the grinding of the herbal drug, with approximately 20 % loss of total sesquiterpenes occurring after 15 to 20 days of powdering [5]. This substantial loss of bioactive constituents can adversely affect the bioactivity of the herbal product. According to the Chinese monograph for Indian costus, the minimum content should be 0.6 % for costunolide and 1.8 % for the combined total of costunolide and dehydrocostus lactone in dried herbal drug form [5]. Therefore, the means of CO and CO + DC in the Indian costus powder and root samples were compared using the independent samples *t*-test. The mean CO levels in both the powder (0.99 %) and root samples (1.0 %) exceeded the 0.6 % threshold, while the mean for CO + DC was below the 1.8 % limit (1.69 % in powder and 1.72 % in root samples). The statistical analysis indicated no significant difference in the content of CO and total sesquiterpene lactones between the powder and root samples (Table 3). This finding may support claims made by herbal shops that powdered herbal drugs are freshly ground and sold within a few days of preparation. Additionally, it suggests that the packaging of powdered Indian costus products is suitable for short-term storage. However, the stability of the powdered samples can vary considerably based on factors such as weather, packaging, and storage conditions.

**Table 3:** Independent samples *t*-test of CO and CO + DC in powder and root samples of Indian costus.

Independent Samples Test										
		Levene's Test for Equality of Variances		*t*-test for Equality of Means						
		F	Sig.	t	df	Sig. (2-tailed)	Mean Difference	Std. Error Difference	95 % Confidence Interval of the Difference	
									Lower	Upper
CO	Equal variances assumed	0.753	0.390	−0.282	47	0.779	−0.03277	0.11625	−0.26664	0.20109
	Equal variances not assumed			−0.277	36.409	0.783	−0.03277	0.11814	−0.27228	0.20674
CO + DC	Equal variances assumed	0.395	0.533	−0.159	47	0.875	−0.02963	0.18675	−0.40532	0.34606
	Equal variances not assumed			−0.158	38.289	0.875	−0.02963	0.18698	−0.40807	0.34881
	Form	N	Mean			Std. Deviation			Std. Error Mean	
CO	Powder	30	0.9893			0.38546			0.07037	
	Root	19	1.0221			0.41364			0.09489	
CO + DC	Powder	30	1.6930			0.63560			0.11604	
	Root	19	1.7226			0.63910			0.14662	

**Fig. 6:**
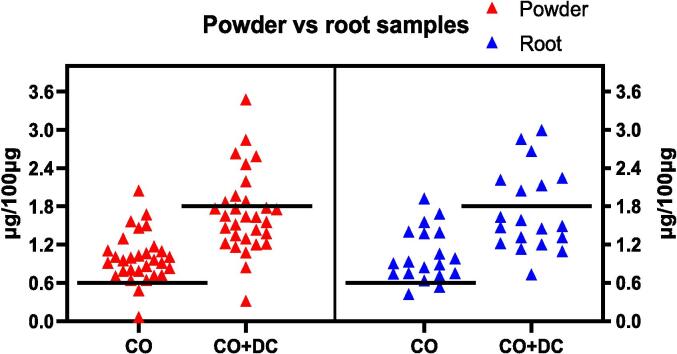
A nested graph representation of the CO and CO + DC content in powder and root samples of Indian costus.

On an individual basis, all powder samples except two exhibited CO concentrations exceeding the minimum limit, while only nine samples passed the 1.8 % limit of CO + DC (Fig. 6). Similarly, all root samples except two had CO concentrations above the minimum threshold, but only seven root samples passed the 1.8 % CO + DC limit. (Fig. 6). While most of the powder and root samples satisfied the CO requirement, many failed to meet the CO + DC limit, indicating that DC content plays a significant role in the overall sesquiterpene lactone concentration. As a result, many of the Indian costus samples analysed may not be effective for their intended medicinal purposes due to insufficient levels of bioactive compounds. This highlights the importance of quick, cost-effective analytical methods for determining the key bioactive constituents in herbal products. Standardised products could provide a reliable solution to ensure quality and dosage consistency in herbal medicines.

#### 3.4.3 Correlation and principal component analysis

**Fig. 7:**
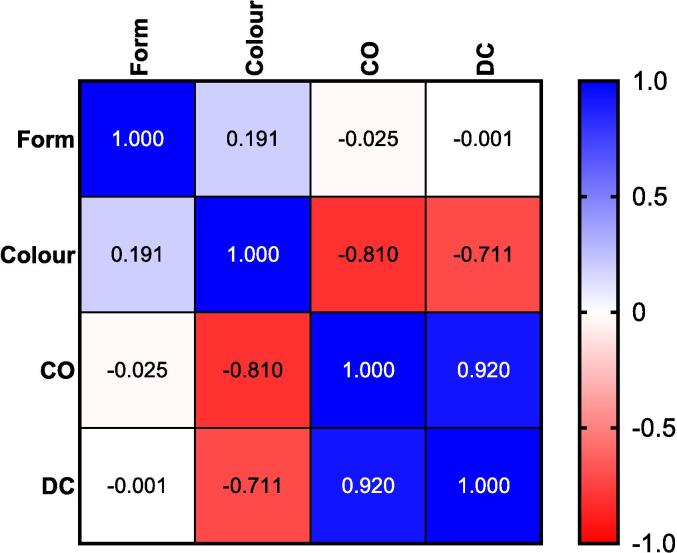
An illustrative heatmap of Spearman's correlation.

Before conducting the statistical correlation analysis, several preliminary tests were performed to determine the appropriate model. Grubbs' test was used to identify any outliers, and both the Kolmogorov-Smirnov and Shapiro-Wilk tests were applied to assess data normality. Grubbs' test did not reveal any significant outliers (P > 0.05), while the normality tests indicated that the data significantly deviated from a normal distribution (P < 0.05). Consequently, bivariate Spearman's correlation analysis (N = 49) was employed to examine relationships between variables. The Spearman's analysis revealed a strong positive correlation between CO and DC. Additionally, an inverse correlation was observed between sample colour and sesquiterpene lactone content (CO and DC), with lighter-coloured samples containing higher levels of these compounds. This finding aligns with Dioscorides' ancient assertion that the highest-quality costus root is light in colour [3]. Moreover, Spearman's analysis confirmed no significant correlation between the form of the herbal product and the sesquiterpene lactone content. The results of the Spearman's correlation are presented in Table 4 and Fig. 7.

**Table 4:** Bivariate Spearman's correlation analysis of Indian costus samples.

Variables	Spearman Correlation	Sig. (2-tailed)	95 % Confidence Intervals (2-tailed)^a^	
			Lower	Upper
Form − Colour	0.191	0.188	−0.103	0.455
Form − CO	−0.025	0.864	−0.312	0.266
Form − DC	−0.001	0.992	−0.303	0.275
Colour − CO	−0.810	< 0.001	−0.891	−0.681
Colour − DC	−0.711	< 0.001	−0.837	−0.548
CO − DC	0.920	< 0.001	0.853	0.953

a Estimation is based on Fisher's r-to-z transformation.

**Fig. 8:**
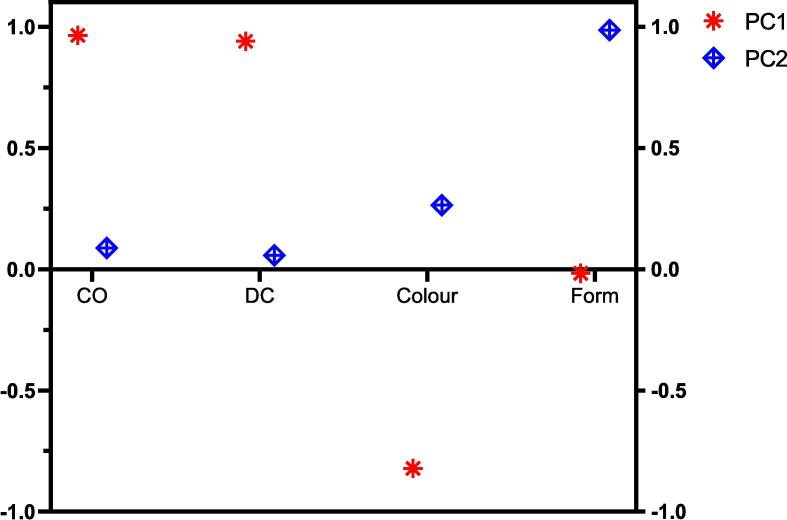
PCA rotated component matrix of costus samples.

Principal component analysis (PCA) was employed to summarise the data and explore the relationships between variables. The scree plot indicated two principal components. The loading of these components for the costus samples accounted for a cumulative variance of 88.56 %, with the first component (PC1) explaining 62.40 % of the variance and the second component (PC2) accounting for 26.16 %. The Kaiser-Meyer-Olkin (KMO) measure and Bartlett's test demonstrated sampling adequacy (0.615) and statistical significance (p < 0.001), indicating that the correlation matrix is significantly different from an identity matrix (Table 5). This revealed that there are sufficient correlations between the variables, making it appropriate for factor analysis. Sesquiterpene lactones (CO and DC) and colour were strongly associated with PC1, while the form of the herbal sample showed a strong loading on PC2. The PCA results confirmed the positive correlation between CO and DC and the inverse correlation between colour and sesquiterpene lactones (Fig. 8). The form of the herbal sample showed no significant correlation with other variables.

**Table 5:** PCA analysis, KMO and Bartlett's test.

Variables	PC1	PC2	KMO and Bartlett's test		
CO	0.964	0.087	Kaiser-Meyer-Olkin Measure of Sampling Adequacy.		0.615
DC	0.94	0.057	Bartlett's Test of Sphericity	*X^2^*	119.1
Colour	−0.822	0.264		df	6
Form	−0.017	0.986		Sig.	<0.001
***Individual variance (%)***	62.40	26.16			
***Cumulative variance (%)***	62.40	88.56

#### 3.4.4 K-mean cluster analysis

**Fig. 9:**
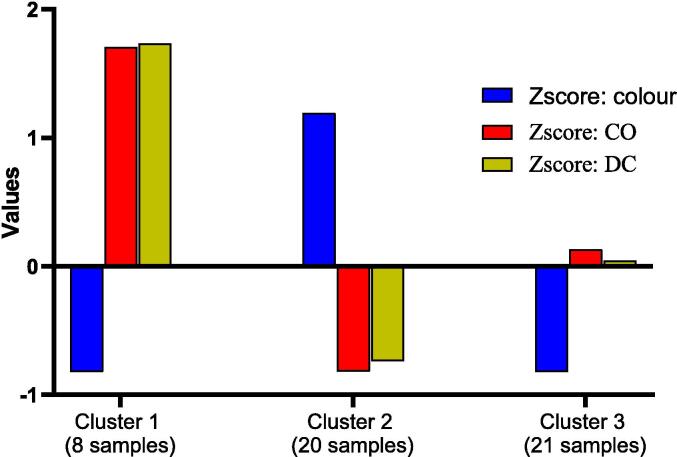
K-mean analysis with cluster loading for the dependent variables in Indian costus samples.

The samples (N = 49) were distributed into three groups (Fig. 9) as clusters: cluster 1 (8 samples), cluster 2 (20 samples), and cluster 3 (21 samples). Cluster 1 represents the Indian costus samples with light colour and the highest concentrations of CO and DC. Cluster 2 consists of dark-coloured samples with the lowest amounts of CO and DC. The third cluster comprises samples with light colour and medium concentrations of sesquiterpene lactones. The K-means analysis further confirms the inverse correlation between colour and sesquiterpene lactones. Although costunolide is a colourless crystalline compound and dehydrocostus lactone is a white powder [4], the correlation between light colour and sesquiterpene lactones in Indian costus can be attributed to complex interactions between biosynthetic pathways, environmental influences, and genetic factors. Furthermore, the presence of colourless or light-coloured compounds, such as sesquiterpene lactones, can “dilute” the overall colour of the sample. Further research could enhance our understanding of these relationships and how they impact the efficacy and quality of herbal products.

## 4 Conclusion

This study resulted in the development of a fast, green, and cost-effective extraction and analysis method for the quantification of sesquiterpene lactones from Indian costus. The UPLC-MSMS method developed for quantifying CO and DC in Indian costus is a rapid, eco-friendly, safe, and cost-effective approach that uses ethanol and water as the mobile phase with a minimal flow rate. The UB method has an advantage over the UH method due to its larger capacity, making it an excellent option for high-throughput extraction. Among the investigated extraction solvents, aqueous ethanol (50 %) exhibited the best extraction efficiency for CO and DC. The developed extraction and analysis method was practically applied to 49 Indian costus samples collected from herbal shops in Saudi Arabia. The statistical analysis revealed significant variations among the samples concerning their CO, DC, and CO + DC concentrations. Most samples contained CO concentrations above the minimum limit (0.6 %) stated by the Chinese monograph; however, only a few passed the 1.8 % limit for CO + DC. Correlation analysis revealed an inverse correlation between the colour of the powder samples and sesquiterpene lactones, as well as a neutral correlation between the herbal form and sesquiterpene lactones. A further in-depth evaluation is necessary to explain the factors involved in the colour of the samples and their effects on sesquiterpene lactone concentrations.

The bioactivity of Indian costus depends on the content of the bioactive sesquiterpene lactones, and a deficiency of these compounds can lead to a loss of efficacy. Therefore, standardised herbal products with minimum limits for bioactive constituents are one appropriate solution for controlling the quality and dosage of herbal products. Standardisation requires fast and cost-effective extraction and analytical methods to determine the major bioactive ingredients in herbal drugs. This study contributes to the standardisation and quality control of herbal products through the extraction and analysis method developed for this purpose. The method may also have several research applications for investigating and quantifying sesquiterpene lactones in herbal, pharmaceutical, food, and cosmetic products. However, factors such as solvent concentration, extraction time, and temperature require optimisation for optimal extraction. Furthermore, the analytical method is limited to quantifying two major bioactive sesquiterpene lactones, while other phytoconstituents may also contribute to the overall bioactivity.

Ethics approval

Although this study does not involve the use of any animal or human data or tissue, experiments on botanical samples in Saudi Arabia require obtaining ethical approval from a local ethics committee registered with the National Committee for Biomedical Ethics (NCBE). Therefore, ethical approval was obtained from Imam Abdulrahman Bin Faisal University Standing Committee for Research Ethics on Living Creatures (SCRELC) with the number IRB-2024–05-548.

## CRediT authorship contribution statement

**Mohammed Aldholmi:** Writing – review & editing, Writing – original draft, Visualization, Validation, Software, Resources, Project administration, Methodology, Investigation, Formal analysis, Data curation, Conceptualization.

## Declaration of competing interest

The authors declare that they have no known competing financial interests or personal relationships that could have appeared to influence the work reported in this paper.
